# Subacute ruminal acidosis in cattle: A critical review of clinical management

**DOI:** 10.1007/s11259-025-10842-5

**Published:** 2025-08-06

**Authors:** Georgios Christodoulopoulos

**Affiliations:** Department of Animal Science, Agricultural University of Athens, Athens, Greece

**Keywords:** Subacute ruminal acidosis, Dairy cattle, Beef cattle, Rumen pH, Clinical signs, Diagnosis, Prevention, Management

## Abstract

Subacute Ruminal Acidosis (SARA) is a prevalent metabolic disorder in high-producing dairy and beef cattle, resulting from prolonged ruminal pH depression due to the provision of excessive amount of rapidly fermentable carbohydrates combined with inadequate fiber intake. The condition impairs rumen function, reduces feed efficiency, and negatively affects animal health and productivity. This review critically examines current approaches to the diagnosis and clinical management of SARA, with particular emphasis on recent advances in diagnostic methods, including computerized rumen mucosa colorimetry applied at the slaughterhouse for herd-level assessment. Nutritional strategies are discussed in detail, focusing on Total Mixed Ration (TMR) formulation, the maintenance of an appropriate forage-to-concentrate ratio, and the inclusion of sufficient potentially fermentable Neutral Detergent Fiber (pfNDF) to support rumen motility and microbial balance. Practical tools such as mathematical models for calculating optimal dietary roughage content are also presented. By integrating current research findings with clinically relevant recommendations, this review would contribute to veterinarians, nutritionists, and livestock producers comprehension of improving rumen health, optimizing animal performance, and preventing SARA under commercial feeding conditions.

## Introduction

Subacute ruminal acidosis (SARA) is a common metabolic disorder in cattle, characterized by prolonged periods of depressed ruminal pH. It is primarily associated with diets high in concentrates and low in digestible fiber, especially potentially fermentable neutral detergent fiber (pfNDF). SARA is a major concern in intensive cattle production systems and, to a lesser extent, in pasture-based systems. It contributes to reduced feed efficiency, decreased milk yield, increased risk of progression to acute ruminal acidosis (ARA), and higher veterinary costs (Church 1991; Bramley et al. 2013; Thonney and Hogue 2013; Kovács et al. 2020; Srivastava et al. 2021; Lorenz 2022; Voulgarakis et al. 2024a).

Across European cattle clinics, SARA is commonly defined as a condition where the ruminal pH stays below 5.8 for at least four hours per day, without dropping below 5.5. This definition has been largely adopted following the meta-analysis of Zebeli et al. (2008), which concluded that ruminal pH should not remain below 5.8 for more than 5.24 h daily to mitigate the risk of SARA. Although the condition has been primarily investigated in dairy cattle, emerging data indicate that similar clinical and subclinical presentations can occur in small ruminants as well (Voulgarakis et al. 2024b).

Unlike ARA, which causes rapid and severe ruminal mucosal necrosis (Steele et al. 2009), SARA is typically associated with milder, non-necrotic mucosal changes (Voulgarakis et al. 2024a, b). Differentiating between SARA and ARA remains critical for prognosis and the selection of appropriate therapeutic strategies (Krause and Oetzel 2006; Voulgarakis et al. 2023).

Earlier definitions of SARA were often inconsistent and overlapped with those of ARA. For example, Kleen et al. (2003) described SARA using a pH threshold of ≤ 5.5, which is now typically considered characteristic of acute acidosis. Likewise, Jaramillo-López et al. (2017) proposed that SARA occurs at ruminal pH values between 5.5 and 5.0, further contributing to historical confusion.

This review aims to clarify the clinical and associated signs of SARA and provide critical information on its pathogenesis and diagnosis. Emphasis is placed on practical, clinically relevant experiences derived from herd-level investigations and rumen mucosa assessment. In particular, this review highlights mathematical approaches to estimate optimal dietary fiber levels for SARA prevention and focuses the discussion on aspects supported by direct clinical expertise, deliberately avoiding topics grounded solely in secondary literature.

## Terminology

Over the years, several terms have been proposed in the literature to describe moderate forms of ruminal acidosis that are neither overtly acute nor entirely physiological. This diversity in nomenclature reflects differences in interpretation regarding the duration, severity, and especially the clinical expression of the disorder.

Kleen et al. (2003) comprehensively reviewed the various terms used historically and in contemporary research to describe these conditions. These include “subacute ruminal acidosis” (Garrett 1996; Nordlund et al. 1995; Stock 2000), “chronic rumen acidosis” (Slyter 1976; Garry 2002), “subclinical rumen acidosis” (Møller 1993; Nocek 1997), “chronic-latent acidosis” (Dirksen 1985; Gäbler 1990), and “latent acidotic stress” (Rossow 1984). Some authors have even proposed a differentiation between “chronic/subclinical” and “subliminal acidosis” (Owens et al. 1998). As Kleen et al. (2003) concluded, the term “subacute ruminal acidosis” (SARA) is most appropriate, as the condition has measurable physiological impacts and clinically detectable consequences, even if these signs are mild or intermittent.

The term “subclinical ruminal acidosis” has recently re-emerged in the literature, with some researchers suggesting it better describes cases with subtle manifestations or those identified primarily through rumen pH monitoring (Golder and Lean 2024). Specifically, Golder and Lean (2024) propose using “subclinical ruminal acidosis” instead of “subacute” to reflect the often inapparent clinical signs in cattle and to emphasize the need for a broader diagnostic approach beyond ruminal pH. They argue that while “subacute” implies a specific timeframe, “subclinical” more accurately captures the nature of the condition in most dairy cows.

Nonetheless, the designation “subclinical ruminal acidosis” implies the absence of clinical signs, which does not fully reflect the clinical presentation in cattle. Furthermore, this term is less appropriate for small ruminants, where SARA frequently presents with obvious diarrhea, a clinical sign that stands out due to the normally solid consistency of their feces. Using “subclinical” in such cases may downplay the clinical relevance of the disorder. While “subacute” could be criticized for suggesting a specific temporal framework, this limitation is of relatively minor concern in clinical livestock practice, where the duration of subacute conditions is not strictly defined.

Despite ongoing discussions, the term SARA continues to be widely used and accepted in the scientific community. A PubMed search in 2025 identified at least 11 publications in cattle medicine using the term “subacute ruminal acidosis,” highlighting its continued relevance.

Altogether, the term SARA is historically grounded and clinically appropriate, emphasizing that affected animals show clinical signs and should not be classified as subclinical.

## Prevalence

SARA is increasingly recognized as a significant challenge in both the dairy and beef industries, even in well-managed, high-producing herds. However, epidemiological data on its prevalence remain limited due to constraints in animal health monitoring resources, as well as inconsistencies in study methodologies and the limited scope of small-scale investigations.

In dairy herds, prevalence rates vary widely, with studies reporting between 0% and 40% of cows affected on individual farms (Garrett et al. 1997; Kleen 2004; Kleen et al. 2009, 2013). A survey of 15 Holstein herds in the United States found SARA in 19% of early lactation cows and 26% of mid-lactation cows, with over 40% of cows affected in one-third of the herds (Garrett et al. 1997). In Europe, Kleen et al. (2009) reported a 13.8% prevalence in the Netherlands, with farm-specific rates ranging from 0 to 38%. A study in Germany found prevalence rates of 11% in early lactation cows and 18% in mid-lactation cows (Kleen 2004). Similarly, a survey in Northern Germany involving 315 cows from 26 farms reported a SARA prevalence of 20%, with substantial variability among farms (Kleen et al. 2013).

In beef cattle, particularly in feedlots, SARA prevalence is generally higher due to dietary transitions to high-energy, grain-based diets (Ma et al. 2021; Simanungkalit et al. 2023). However, robust epidemiological data for beef cattle remain scarce. A study in Egypt examining two fattening farms reported SARA incidence rates ranging from 32.5 to 37.7% (Attia 2016).

## Pathogenesis

In the rumen, sugars and starches are fermented into volatile fatty acids (VFAs), primarily acetate, propionate, and butyrate, which serve as key energy sources for the host animal (Dijkstra 1994; Kovács et al. 2020). In the context of SARA, VFAs are also referred to as short-chain fatty acids (SCFAs), as they consist of fatty acids with fewer than six carbon atoms (Mathiesen et al. 2005; Shen et al. 2019). Under normal conditions, rumen pH is maintained within an optimal range of 5.8 to 6.8 (Shen et al. 2019). However, when digestible fiber content falls below 25% and rapidly fermentable carbohydrate intake exceeds 50%, excessive VFAs accumulation leads to a sustained drop in ruminal pH below 5.8, predisposing cattle to SARA (Enemark et al. 2002; Kovács et al. 2020; Srivastava et al. 2021).

Early characterizations of microbial shifts associated with ruminal acidosis date back to studies from the 1960s. According to the understanding at that time, a drop in ruminal pH below 5.5 suppresses Gram-negative bacterial populations while promoting the proliferation of acid-tolerant Gram-positive cocci, notably *Streptococcus bovis*, *Streptococcus equinus*, and *Streptococcus gallolyticus*. These species produce significant quantities of lactic acid, which further decreases ruminal pH and may push the condition toward acute ruminal acidosis (ARA). In addition, the accumulation of lactic acid increases the osmotic pressure within the rumen contents, thereby exacerbating ruminal dysfunction (Kaufmann and Rohr 1967).

More recent research has refined this understanding through the application of multi-omic approaches, offering a broader and more nuanced view of the rumen microbiome. These studies reveal that a significant portion of ruminal microbes remain uncultured and uncharacterized, suggesting that additional taxa beyond the traditionally recognized lactic acid producers may contribute to acidosis pathogenesis. Moreover, microbial communities operate as complex ecosystems exhibiting metabolic redundancy, which complicates efforts to identify singular microbial signatures diagnostic of ruminal acidosis. Individual animals harbor unique core and noncore microbiomes that influence their susceptibility to ruminal disorders, underscoring the need for personalized approaches in diagnosis and management (Jami and Mizrahi 2012; Zehavi et al. 2018; Golder and Lean 2024).

**Fig. 1:**
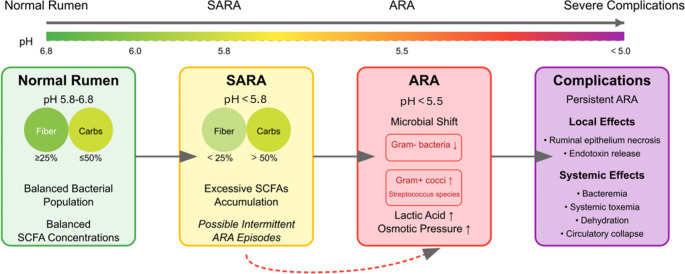
Schematic representation of the proposed pathogenesis of ruminal acidosis based on current understanding. This conceptual model illustrates key causes, microbial involvement, metabolic changes, and clinical signs of ruminal acidosis in a simplified manner and does not encompass all possible variations

When ARA persists, severe acidification can lead to ruminal epithelial necrosis, bacteremia, and systemic toxemia due to endotoxin release from lysed Gram-negative bacteria. Increased osmotic pressure results in dehydration and circulatory collapse, which can be fatal (Owens et al. 1998). Notably, cows with SARA often experience intermittent ARA episodes, explaining the diverse clinical presentations observed (Steele et al. 2009). Figure 1 provides a simplified overview of the pathogenesis of ruminal acidosis.

## Causes

SARA is commonly associated with high-concentrate diets but can also occur in pasture-based systems when the intake of non-fiber carbohydrates is elevated (Plaizier et al. 2012; Bramley et al. 2013; Abaker et al. 2017). In both situations, fiber plays a crucial role in maintaining rumen stability. Research from Cornell University has emphasized the significance of a specific fiber fraction known as potentially fermentable Neutral Detergent Fiber (pfNDF), which mainly includes the cellulose and hemicellulose components of NDF. This fraction is essential for supporting healthy rumen fermentation. Tabular values of pfNDF have been developed for the major feedstuffs commonly used in cattle diets (Table 1); however, individual feed test results from the respective farm should be used in preference whenever available. When pfNDF levels are insufficient, rumen microbes ferment starch rapidly, resulting in excessive accumulation of volatile fatty acids, a decline in ruminal pH, and a higher risk of SARA (Hogue 1993; Van Soest 1994; Thonney and Hogue 2013).

**Table 1:** Selected component values in common livestock feeds. [adapted from Van Soest (1994) and Thonney and Hogue (2013) ]

Ingredient		NFC*	CP*	NDF*	pfNDF*
**Forages**					
Alfalfa	Early bloom	27	19	42	19
	Mid bloom	25	17	46	21
	Late bloom	23	12	55	23
Orchard grass	Early bloom	20	10	57	37
	Late bloom	13	8	67	36
Timothy	Late veg.	20	14	55	40
	Early bloom	18	11	61	40
	Late bloom	14	8	68	39
	Seed stage	14	6	72	34
Corn silage, 45% grain		42	9	41	28
Wheat straw		2	3	85	40
**Grains**					
Barley	Heavy	63	13	19	14
	Light	52	14	28	17
Corn		75	10	9	6
Oats, 32 lb/bushel		37	13	42	27
Wheat		69	11	16	10
**By-pr**od**ucts**					
Beet pulp		32	8	54	40
Citrus pulp^a^		44	7	38	32
Corn germ meal		26	27	41	29
Corn gluten feed		18	25	45	40
Cottonseed hulls		0	4	90	50
Dried brewers grains		17	26	46	28
Dried distillers grains		10	26	50	42
Hominy		25	12	55	50
Oat hulls		9	4	78	28
Soy hulls		11	12	70	62
Wheat midds		40	18	37	32
**Protein supplement**					
Soybean meal, 44% CP		28	49	14	9

NFC: Non-Fiber Carbohydrates (includes sugars and starches)

CP: Crude Protein

NDF: Neutral Detergent Fiber

pfNDF: potentially-fermentable NDF

*% of dry matter

^a^15% pectin-like-substances (in Citrus pulp, p-l-s are incorporated into the pfNDF fraction)

In intensive cattle operations, SARA often arises when concentrates are offered separately from roughage, leading to selective feeding behaviors that exacerbate dietary imbalances. Even in total mixed ration (TMR) systems, inadequate straw inclusion or improper mixing can increase ration sorting, reduce roughage intake, and predispose cattle to SARA (Kleen and Cannizzo 2012).

In pasture-based systems, SARA can also develop, particularly when cows graze on lush, early vegetative pastures with high fermentability and low effective fiber content (Bramley et al. 2013). Feeding practices that combine such pasture with concentrate supplementation may increase the risk of acidotic conditions. Additional risk factors include variability in access to pasture and supplements among individual cows (Bramley et al. 2012, 2013).

## Clinical signs

To date, the only clinical signs consistently reported in cases of SARA are changes in fecal characteristics (Mutsvangwa et al. 2002; Krause and Oetzel 2005; Tajik and Nazifi 2011). However, these changes are neither pathognomonic nor reliable indicators of ruminal acidosis, as their predictive value has been shown to be low (Bramley et al. 2013). This limitation is one of the main reasons why many researchers support the continued use of the term “subclinical ruminal acidosis” to describe the condition (Golder and Lean 2024).

Fecal alterations observed in SARA typically include mild diarrhea and changes in color, with feces appearing lighter and more yellowish (Rossow et al. 1984; Dirksen 1985; Oetzel 2000; Garry 2002; Kleen et al. 2003; Nordlund 2003; Krause and Oetzel 2006; Enemark 2008; Li et al. 2012; Abdela 2016; Chaudhry et al. 2018). Additionally, foam may be present (Abdela 2016). The fecal pH is typically slightly acidic, and the odor is often described as sweet–sour (Dirksen 1985; Oetzel 2000). The particle size of ingesta in the feces tends to be larger than normal—around 1–2 cm instead of the typical less than 0.5 cm—and undigested whole cereal grains may also be observed (Garry 2002). These changes are generally transient (Abdela 2016).

Two main mechanisms are believed to explain these fecal changes:

- Post-ruminal fermentation: The accelerated passage of fermentable carbohydrates into the intestines leads to microbial fermentation beyond the rumen, contributing to fecal foam and altered consistency (Bolton and Pass 1988; Owens et al. 1998; Garrett et al. 1999; Oetzel 2000).
- Increased osmolarity of ruminal contents: The elevated osmolarity draws water into the intestinal lumen, leading to looser, more liquid feces (Garry 2002).

## Associated signs

In addition to the changes in fecal characteristics, several other signs, although not strictly clinical or not yet proven to be directly caused by SARA, are nonetheless frequently observed in affected animals. These signs may support the overall clinical impression of SARA at the herd level.

Milk fat depression has been considered a key indicator of SARA in many studies (Kleen et al. 2003; Nordlund 2003; Krause and Oetzel 2006; Enemark 2008; Shingfield et al. 2010; Li et al. 2012; Abdela 2016). The reduction in rumen pH disrupts normal fermentation, typically by decreasing acetate and increasing, in particular, butyrate concentrations. The resulting lower acetate availability limits *de novo* fatty acid synthesis in the mammary gland. Furthermore, altered rumen biohydrogenation under acidic conditions leads to the accumulation of trans-fatty acid intermediates—particularly trans-10, cis-12 conjugated linoleic acid (CLA)—that inhibit the expression of lipogenic enzymes and reduce milk fat synthesis (Bauman and Griinari 2003; Shingfield et al. 2010). The resulting inhibition of milk fat synthesis ultimately lowers milk fat content, leading to economic losses for dairy producers (Chaudhry et al. 2018; Voulgarakis et al. 2023). According to Nordlund (2004), a milk fat percentage below 2.5% in at least 10% of cows in a Holstein herd may indicate SARA.

Additional signs associated with SARA include reduced feed intake, decreased milk yield, weight loss, parakeratosis, and liver abscesses. The pathogenesis of these symptoms has been extensively discussed in the literature (Kleen et al. 2003; Hossain 2020). These signs are more commonly linked to acute acidosis, as their pathogenesis involves inflammation, which can occur in farms where SARA episodes progress to ARA.

A possible association between SARA and laminitis has been proposed by several authors (Nocek 1997; Westwood et al. 2003; Abdela 2016; Hossain 2020). Although laminitis is a well-recognized and economically significant condition in dairy cows, its pathophysiological link to SARA remains under debate. Early discussions among clinicians often relied on extrapolations from equine medicine, despite the clear differences in pathophysiology between horses and cattle. Moreover, several researchers have questioned the strength of this association, emphasizing that the exact etiology of bovine laminitis and its relationship with ruminal acidosis remain unresolved (Frankena et al. 1992; Bargai and Levin 1993; Lischer and Ossent 1994; Nordlund et al. 1995; Kleen et al. 2003).

From a different perspective, one may also consider whether lameness itself could predispose cows to SARA. Lame cows tend to shorten their time spent feeding on pasture and may rely more heavily on provided concentrates and by-products, which could increase the risk of ruminal acidosis. Under this view, the findings of Bramley et al. (2013), where dairy herds with a higher risk of lameness also showed a greater risk of acidosis, might reflect an effect of lameness on the occurrence of SARA rather than the reverse.

## Lesions

Regarding pathology, SARA induces slight changes to the rumen wall, with the epithelium remaining intact (Voulgarakis et al. 2024a, b). In contrast, ARA is characterized by visible inflammation, lymphocyte infiltration, detachment of the keratinized epithelium, and occasionally parakeratosis (Steele et al. 2009).

A dark coloration of the ruminal epithelium has been widely reported in association with SARA (Alhidary et al. 2016; Voulgarakis et al. 2024a, b). Some researchers have linked this grey-to-dark discoloration to parakeratosis (Gäbler 1990; Enemark 2008; Steele et al. 2009). However, our findings (Voulgarakis et al. 2024a, b) did not reveal any evidence of parakeratosis in cases where discoloration from grey to black was observed. Based on these results, we speculate that the parakeratosis described by other researchers (Gäbler 1990; Enemark 2008; Steele et al. 2009, 2011) pertains to findings associated with ARA. Additionally, our study noted that the darkest coloration was limited to the keratinized layer of the epithelium, suggesting that the discoloration results from the effect of rumen pH on this layer (Voulgarakis et al. 2024a, b).

Histological examination in our research revealed an increase in the thickness of the non-keratinized epithelium, which led to a corresponding increase in total epithelium thickness in animals experiencing prolonged SARA. This phenomenon may be attributed to the lower rumen pH, which potentially accelerates the turnover rate of the keratinized epithelium. Consequently, this necessitates increased production of non-keratinized epithelium to sustain the formation of new keratinized layers (Voulgarakis et al. 2024a).

## Diagnosis

In SARA, the absence of pathognomonic symptoms complicates diagnosis (Nocek 1997; Tajik and Nazifi 2011; Snyder and Credille 2017). In addition, there is currently no consensus on a routine detection method for the disorder in practice.

In terms of ruminal clinical medicine, direct measurement of ruminal pH has traditionally been regarded as the most accurate method for detecting SARA, given its ability to directly reflect the acid–base balance in the rumen (Enemark 2008; Humer et al. 2018). Since rumen pH fluctuates throughout the day (Dado and Allen 1993; Steele et al. 2009), multiple measurements are needed to confirm whether pH stays between 5.5 and less than 5.8 for at least four hours per day. For herd-level screening, sampling 12 cows is typically sufficient to assess the status of the group (Kleen et al. 2003; Nordlund 2003).

The best way to evaluate rumen pH fluctuation is to insert a pH probe directly into rumen digesta and record pH in real-time (Penner et al. 2006). Indwelling rumen pH devices are commercially available and come with a built-in data logger and wireless communication technology (Penner et al. 2006). However, the use of these devices on farms is still very limited due to costs, but most importantly due to their short lifespan of roughly six months and the considerable drift they experience, which reduces their accuracy (Kaur et al. 2010; Han et al. 2022).

In the absence of rumen pH devices, the standard clinical method involves taking a rumen sample to measure pH on the farm using electronic pH meters, which are now highly accurate and relatively inexpensive. Sampling should be timed for when ruminal pH is at its lowest. Consequently, sampling is recommended within 2–4 h after the concentrate meal in herds fed separate components and within 5–8 h in TMR fed herds (Nordlund et al. 1994; Kleen et al. 2003). Ideally, sampling multiple times during the day increases accuracy, though it is not always practical.

**Fig. 2:**
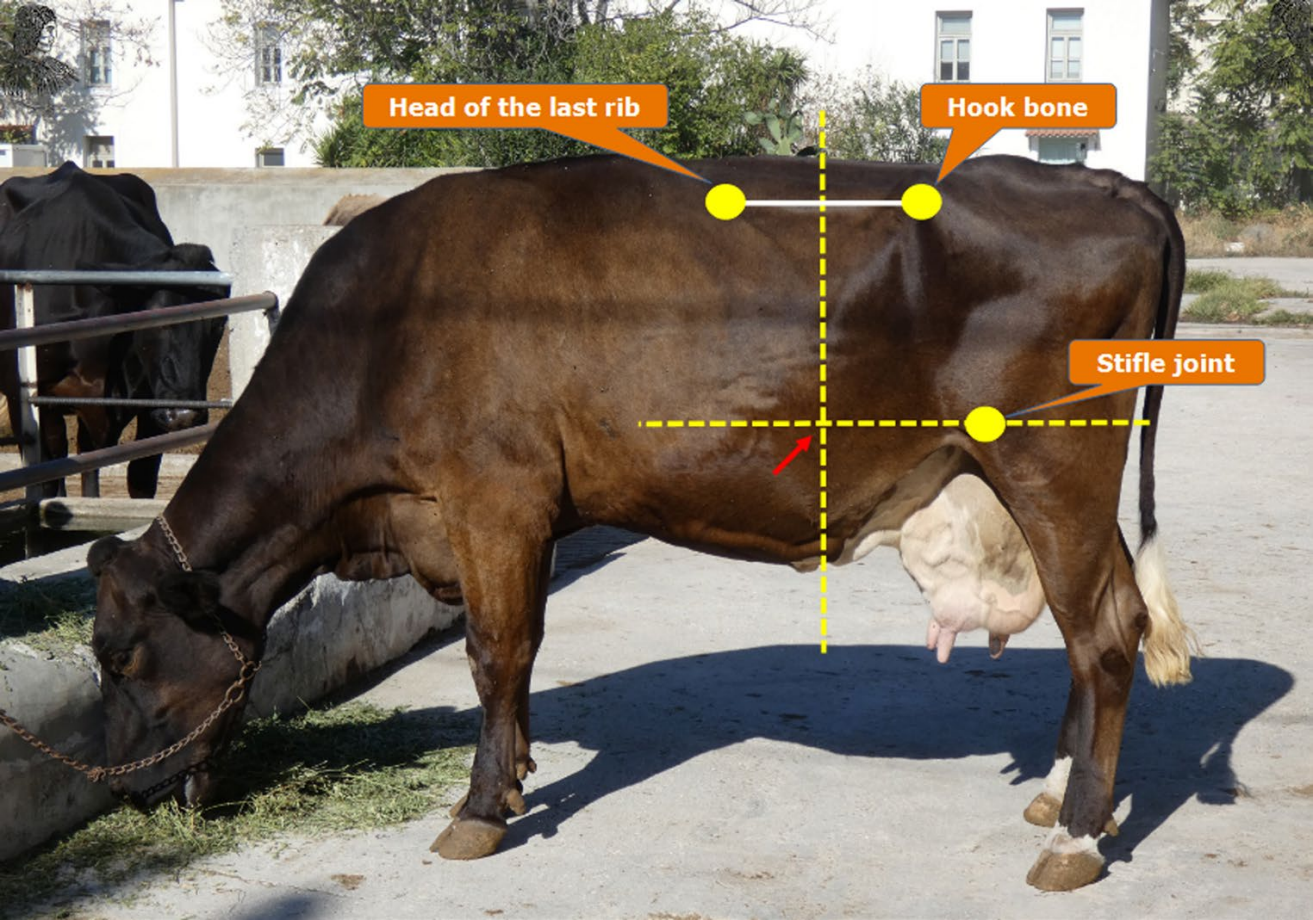
The proposed site for rumenocentesis (arrow) is located on the left side of the animal, at the intersection of a vertical line drawn through the midpoint of the segment connecting the last rib head and the hook bone (tuber coxae), and a horizontal line passing through the stifle joint. Although this vertical line often intersects the lowest point of the paralumbar fossa, we do not recommend using this specific point as a landmark, as it may be difficult to identify in overconditioned or heavily loaded animals. Rumenocentesis is an invasive procedure with animal welfare implications and should be performed only when clearly justified for diagnostic or research purposes

In addition to the need for strict timing, the choice of sampling method, whether esophageal tubing or rumenocentesis, was previously a major concern, although this issue has largely been addressed in recent years. While saliva contamination was once a drawback of esophageal tubing, modern sealed devices now effectively prevent it, making this method a safe and accurate option for rumen fluid collection (Geishauser et al. 2012). Rumenocentesis is also an effective alternative but is invasive and may raise welfare concerns. In our practice, for rumenocentesis, we use three reference points: the last rib, the hook bone, and the stifle joint. The correct site is located on the left side of the animal, at the intersection of a vertical line drawn through the midpoint of the segment connecting the last rib head and the hook bone, and a horizontal line passing through the stifle joint (Fig. 2). Similar anatomical points for performing rumenocentesis have been suggested by other researchers (Nordlund and Garrett 1994; Duffield et al. 2004). Although some online sources suggest needle insertion in the lower part of the left paralumbar fossa, we advise against this approach, as the fiber layer in the rumen here frequently obstructs the needle, compromising sample collection.

Despite its widespread use, the diagnostic value of ruminal pH is increasingly questioned. Its sensitivity and repeatability are limited, especially when assessed in individual cows and interpreted without a thorough clinical examination. Additionally, sampling time and variation in feeding practices across farms can significantly influence pH values, making single-point measurements unreliable (Jonsson et al. 2019). The common practice of applying fixed pH thresholds (e.g. 5.5 to 5.8 for SARA diagnosis) without considering clinical or management data has also been criticized as a misuse of early field-based screening tools. These thresholds were originally intended for herd-level interpretation in conjunction with physical examination and herd history (Golder and Lean 2024). Over-reliance on pH as a diagnostic “gold standard” neglects these limitations and may lead to underdiagnosis or misclassification. More robust diagnostic strategies, including volatile fatty acid (VFA) profiles, dry matter intake (DMI), milk performance records and bunk management, are therefore increasingly advocated to enhance accuracy (Jonsson et al. 2019; Golder and Lean 2024).

Given the limitations and declining diagnostic value of rumen pH measurement when used alone, the identification of practical, reliable biomarkers for early SARA detection remains a significant challenge for dairy practitioners (Morar et al. 2022). Indirect parameters proposed as possible predictors of SARA include observation of chewing (ruminating boli per hour) and feeding activities (fluctuating feeding patterns) (Zebeli et al. 2008; Devries et al. 2008), as well as monitoring milk fat, milk fat-to-protein ratio, milk urea nitrogen (MUN) (Enemark et al. 2004; Duffield et al. 2004), the presence of light diarrhea frequently with foam, fecal pH (Kleen et al. 2003; Plaizier et al. 2008), various urine parameters (net acid-base balance, inorganic phosphorus, pH) (Danscher et al. 2015), various blood variables (acute-phase proteins, partial pressure of carbon dioxide, pH, concentrations of Ca, Na, K, Cl) (Gao and Oba 2015; Humer et al. 2018), and ruminal mucosa thickness determined by ultrasound (Danscher et al. 2015). However, due to the limited specificity and precision of these indirect diagnostic measurements, they do not represent powerful diagnostic methods. Therefore, using more than one signal is strongly recommended to reliably identify cows at risk for SARA (Humer et al. 2018), although the optimal combination has not yet been identified or tested.

Recently, our research has investigated whether rumen mucosal color can serve as a diagnostic indicator for subacute ruminal acidosis (SARA). A strong association was confirmed between mucosal discoloration and previous SARA occurrence in beef cattle, supporting its use as retrospective evidence of the disorder. To objectively assess this, we developed “Computerized Rumen Mucosal Colorimetry”, a simple “photo test” involving the capture of a digital image of the rumen mucosa, which is then analyzed using a software application that quantifies color values, including red, green, and blue components (Voulgarakis et al. 2024a). In an ongoing study, this method was applied to slaughtered cattle, and follow-up visits to their farms included rumen pH testing to confirm SARA status. Results showed that assessing a single animal’s mucosa using this technique could identify SARA presence in the herd with 92% sensitivity and 87.5% specificity. These findings support the use of this approach as a practical monitoring tool for herd-level SARA assessment in slaughterhouses.

## Prevention through TMR

Effective management of SARA requires careful formulation and preparation of the TMR, particularly regarding roughage inclusion and processing. Roughage, such as straw, should be chopped to an optimal length to achieve a balance between thorough mixing and adequate rumen stimulation (Grant and Albright 1995). In current practice, the cattle industry typically considers 2.5–5 cm (1–2 inches) as the optimal length. This particle size promotes rumination and saliva secretion, both of which are essential for buffering rumen pH. However, achieving the appropriate chop length alone is not sufficient; improper mixing can result in ration sorting, whereby cows selectively consume the more palatable components while avoiding the roughage. To minimize this risk, mixing times should typically range between 3 and 5 min after the addition of all ingredients, though specific recommendations may vary depending on the mixing equipment used (Grant and Albright 1995). Over-mixing can excessively break down fiber particles, reducing their effectiveness in stimulating rumination, whereas under-mixing can lead to an uneven feed distribution, encouraging selective feeding (Mertens 1997).

Beyond ensuring proper chopping and mixing of roughage, the overall dietary fiber content plays a crucial role in preventing SARA and maintaining optimal rumen function. The National Research Council (2001) recommends that total NDF should comprise 28–34% of the diet, with at least 19–21% of this originating from roughage sources. The roughage-to-concentrate ratio should ideally range between 60:40 and 50:50, depending on production levels, to provide a balance between energy supply and fiber intake (Beauchemin and Yang 2005). Additionally, 20–30% of the total dry matter should consist of physically effective NDF (peNDF), which plays a key role in stimulating chewing activity and saliva production, thereby aiding in rumen pH stabilization; peNDF refers to the fraction of neutral detergent fiber (NDF) with sufficient particle size to promote chewing and contribute to the formation of the floating mat of large particles in the rumen (Mertens 1997; Thonney and Hogue 2013; White et al. 2017). In practical feeding programs, farms commonly include 2–4 kg of chopped roughage per cow per day within the TMR to ensure adequate fiber intake, promoting efficient digestion and overall rumen health.

In our practice, detailed ration formulation begins by calculating the appropriate amount of concentrate mixture (“*Cm”*), ensuring it contains 10–15% pfNDF along with adequate levels of protein, vitamins, and minerals. To achieve the desired pfNDF level, we carefully select concentrate ingredients, considering their pfNDF values based on Table 1.

We then calculate the daily amounts of concentrate and roughage (“*Rg”*) to feed each cow, ensuring the total diet meets both Dry Matter Intake (DMI) and Metabolizable Energy (ME) requirements. For example, the following steps outline how to calculate the daily amounts of dry matter for a lactating dairy cow, given a “*Cm”* with “a” MJ of metabolizable energy and “k”% NDF, and a “*Rg*” with “b” MJ of ME and “q”% NDF.

We first calculate the DMI for the cow if it were to consume only the “*Cm”* or only the “*Rg*”. This is typically expressed as a percentage of the animal’s live weight (W). The amount for “*Cm”* is given by the formula: $\:\frac{120}{k}$% of W (Holland et al. 1990; *PIRSA* 2018). So, in kg, the DMI will be 1.2$\:\frac{W}{k}$

Similarly, if the animal consumes only “*Rg*,*”* the DMI will be 1.2$\:\frac{W}{q}$

**Fig. 3:**
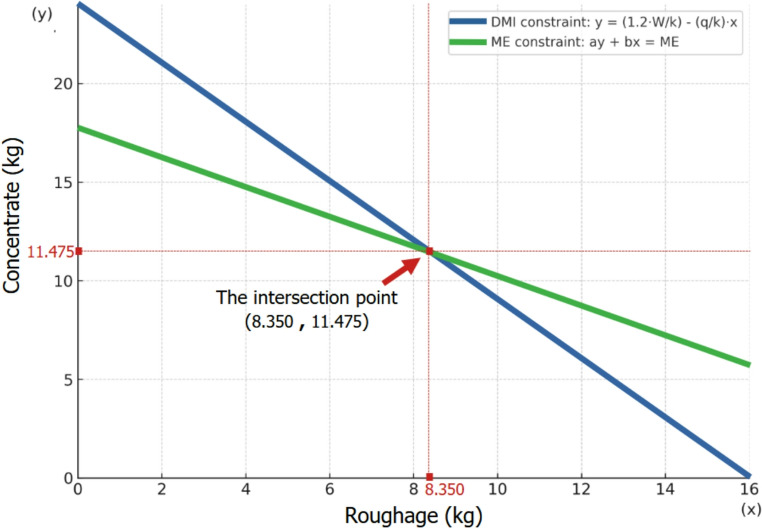
Graphical representation of diet formulation for a lactating dairy cow The blue line represents the Dry Matter Intake (DMI) constraint based on fiber limits, expressed as y=(1.2 W/k)−(q/k)x, where “W” is the cow’s body weight, “k” is the neutral detergent fiber (NDF) percentage of the concentrate, and “q” is the NDF percentage of the roughage The green line represents the Metabolizable Energy (ME) constraint, expressed as ay + bx = ME, where “a” and “b” are the ME contents (in MJ/kg of dry matter) of the concentrate and roughage, respectively, and ME is calculated from milk yield and body weight as ME = 5 L + 0.1 W + 2.85, with “L” being the daily milk yield In the example illustrated, values used are W = 600 kg, L = 30 kg/day, a = 12, b = 9, k = 30%, and q = 45%. The intersection point of the two lines (arrow) defines the exact quantities of concentrate and roughage that simultaneously meet the cow’s energy and fiber intake requirements. By solving the system formed by the two line equations (blue and green lines), the optimal amounts are determined to be 11.475 kg of concentrate and 8.350 kg of roughage on a dry matter basis

On a Cartesian diagram, the possible combinations of “*Cm*” and “*Rg*” amounts are plotted on a line determined by the points (0, 1.2$\:\frac{W}{k}$) and (1.2$\:\frac{W}{q}$, 0). The equation of this line is:

$$y=1.2\;\frac Wk-\frac qkx$$

(A), where y is the DMI of “*Cm”* and x is the DMI of “*Rg”* (both in kg) (Fig. 3).

Next, we calculate the ME requirements of the cow using the following equation:

$$ME\;=\;5\;L\;+\;0.1\;W\;+\;2.85$$

(B), where L is the daily milk yield (kg) and W is the cow’s live weight (kg). The ME can also be calculated using alternative equations or tabular values, depending on specific circumstances.

At the same time, the ME provided by the diet is expressed as:

$$ME\;=\;ay\;+\;bx$$

(C), where y is the amount of “*Cm”* in kg, x is the amount of “*Rg”* in kg, “a” is the ME content of the “*Cm”* per kg, and “b” is the ME content of the “*Rg”* per kg.

By combining equations (B) and (C), we have:

$$ay\;+\;bx\;=\;5\;L\;+\;0.1\;W\;+\;2.85$$

(D) Finally, solving the system of (A) and (D) equations we have the values of x and y, representing the amounts of “*Cm”* and “*Rg”* to be fed daily to meet the cow’s nutritional needs. Figure 4 presents a graphical example for a cow of 600 kg body weight producing 30 kg of milk per day, showing how a concentrate mixture and a roughage of certain characteristics can be combined to meet both energy and fiber intake requirements.

## Prevention through feed additives

Jaramillo-López et al. (2017) have provided a comprehensive review of feed additives used in the prevention of SARA. The supplementation of buffer substances is a widely adopted strategy to mitigate the adverse effects of ruminal acidosis, with inclusion rates in total rations ranging from 0.5 to 2.5%. Commonly used buffer substances include sodium bicarbonate, disodium carbonate, magnesium oxide, potassium carbonate, and anhydrous limestone. These compounds help stabilize rumen pH and counteract acid buildup, thus reducing the risk of SARA.

In addition to buffers, zootechnical additives such as *Saccharomyces cerevisiae* and *Megasphaera elsdenii* have been explored for their potential to enhance rumen function. Furthermore, essential oils such as cinnamaldehyde and eugenol have been investigated as natural alternatives to modulate microbial populations and reduce the incidence of acidosis (Jaramillo-López et al. 2017).

Among feed additives, sodium bicarbonate is the most commonly used for beef cattle, typically included at a rate of 0.5% in the concentrate mix. This supplementation is particularly beneficial when pfNDF content falls below 25%, often due to an increased proportion of carbohydrates aimed at maximizing daily weight gain. A similar supplementation strategy can be applied to dairy cows, as long as it is accompanied by continuous and consistent monitoring of the total ration and milk composition to ensure animal health and maintain productivity.

The last decades, the ionophore monensin has been used in cattle diets to prevent ruminal acidosis. When included at a rate of 400 g per ton of concentrate, monensin was shown to enhance rumen fermentation and reduce the risk of SARA. However, its use in farm animals has been prohibited in the European Union since 2003, prompting the need for alternative feed strategies to maintain rumen health and productivity (European Parliament and Council of the European Union 2003).

## Conclusions

SARA remains a prevalent issue in high-producing dairy and beef herds, primarily resulting from diets rich in fermentable carbohydrates and deficient in potentially-fermentable fiber. Accurate diagnosis using tools such as rumen pH monitoring and rumen mucosa colorimetry is essential for early detection and herd-level assessment.

Prevention relies on appropriate nutritional strategies, including balanced total mixed ration formulation, adequate potentially-fermentable fiber, and targeted use of feed additives. Effective management requires collaboration among veterinarians, nutritionists, and producers to reduce the incidence of SARA and its negative effects on animal health and productivity.

## Data Availability

No datasets were generated or analysed during the current study.
